# GPCRs Revisited: New Insights Lead to Novel Drugs

**DOI:** 10.3390/ph4020244

**Published:** 2011-01-25

**Authors:** Richard M. Eglen, Terry Reisine

**Affiliations:** 1 Bio-discovery, PerkinElmer Life and Analytical Sciences, 940 Winter St., Waltham, MA 02451-1457, USA; 2 Reisine Biotechnology Consultants, 10327 Rossbury Place, Los Angeles, CA 90064, USA; E-Mail: treisine@aol.com (T.R.)

**Keywords:** GPCR, G protein, allosterism, dualsteric, oligomerization, pharmacophore, crystal structure, NMR, DMR, pepducin

## Abstract

GPCRs play a critical role in human physiology and are a prime target for drug discovery globally. Novel insights into the functions of GPCRs are providing unique approaches to modulate these proteins to generate unique drug candidates. Next generation ligands include those with novel pharmacologies such as allosteric regulators as well pepducins, that affect the interaction of GPCRs with G proteins, to either block selective receptor signaling pathways or mimic the actions of intracellular domains of receptors, thereby activating GPCRs to signal selectively to intracellular pathways. We will review these new concepts and then discuss how they may be exploited using modern discovery technologies to provide novel drug candidates for the future.

## 1. Introduction

G protein coupled receptors (GPCRs) constitute one of the largest gene families in the human genome. Due to their critical role in cell-cell communication and as causal agents in disease, they are a target for the development of numerous drugs, many of which have received regulatory approval. Emerging knowledge of GPCR physiology has begun to alter our approach to GPCR drug discovery and is providing insight into how to discover new generations of compounds as human therapeutics [1,2].

GPCRs are integral cell membrane proteins and their association with membrane lipids is critical for conferring, and maintaining, receptor structures required for function. The phospholipid environment controls the inherent flexibility of the receptor mainly due to the structural disorder of the third intracellular loop, a domain known to undergo major conformational changes following agonist binding and to be critical for G protein coupling [3,4]. The pre-requisite for lipid association and structural flexibility hitherto hindered the growth of GPCR crystals needed for X-ray diffraction analysis. This hurdle has recently been overcome with resolution of crystal structures for several GPCRs [4-11], which in turn, allows rational drug design to be employed in GPCR drug discovery and thus novel GPCR drug structures to be identified [12-14].

Unlike most enzymes that are energetically unidirectional (e.g. kinases phosphorylate proteins selectively; proteases hydrolyze selective substrates), GPCR activation can produce a greater diversity of cellular functions as they interact with multiple Guanine nucleotide binding proteins (G proteins) to regulate discrete signaling pathways [15-21]. G proteins bind to allosteric sites on the receptor that are distal to the orthosteric, ligand binding pocket [22]. Furthermore, binding sites exist on the extracellular face of the receptor, distinct from the orthosteric binding domain, representing sites that can be targeted for allosteric drug development. Consequently, a unique opportunity now exists to develop drugs selectively acting at those allosteric sites that modulate GPCRs in a very different manner from archetypal orthosteric ligands.

Some drugs of this type are generically referred to as allosteric modulators, whilst others are referred to as ‘dualsteric’, being molecules that bind both to orthosteric and allosteric sites [23-28]. Recent research has also led to the development of pepducins [29]; cell penetrating peptides that mimic or abrogate GPCR interaction with specific G proteins to induce a discrete cellular response. This new class of GPCR ligands provides several favorable properties over typical orthosteric GPCR agonists, inverse agonists and antagonists.

GPCRs are also known to produce constitutive activity, that is activity in the absence of endogenous ligand or agonist [1,2,29-34]. Constitutive activity may be considered to be an equilibrium state of a GPCR between its ground state of no activity and its fully activated state. Agonists can still bind to constitutively active GPCRs to produce further agonism. However, because of the high basal activity, some compounds can bind to constitutively active receptors and produce opposite responses to agonists. For example, if an agonist at a particular GPCR stimulates an increase in Ca^++^ mobilization and the constitutively active receptor has high basal Ca^++^ mobilization, some ligands are able to bind to this receptor to reduce basal Ca^++^ mobilization down towards the ground state. These ligands have been referred to as ‘inverse agonists’. In many cases, these ligands were originally characterized as antagonists on non-constitutively active GPCRs and their inverse agonism is primarily seen when they interact with constitutively active GPCRs. The existence of constitutive activity has required a reconsideration of how we think about GPCR antagonists. Antagonists are classically considered compounds that bind to GPCRs to block binding and activation by agonists, but produce no activity of their own. In fact, compounds that block both agonist and inverse agonist binding but produce no response on GPCRs are now referred to as ‘neutral antagonists’.

Constitutive activity of GPCRs is believed to be due to their formation of oligomers in addition to single unit assemblies [29-34]. In many immortalized cell systems GPCRs are over-expressed, and thus form homodimers. They may also form heterodimers in which two different GPCRs associate. In some cases dimers exhibit different pharmacological properties than seen with monomers. Notably they may differ in agonist binding due to the formation of a unique orthosteric binding domain. Previously, dimers were considered to only exist in artificial cell systems classically used in GPCR drug discovery. However, emerging evidence suggests that GPCR oligomerization also occurs in endogenous tissues [31]. Consequently, discovery programs aimed at targeting GPCR oligomerization may result in drugs selective for discrete GPCR oligomers, providing unique, and potentially desirable, pharmacological properties in contrast to compounds targeting receptor monomers only.

This review will focus on these new avenues of GPCR drug discovery. We also describe briefly the scientific basis of discoveries leading to these new fields of drug discovery. Finally, we provide information on the potential of novel drugs, as well as new technologies used to screen for this new generation of GPCR compounds.

## 2. Crystal Structure and Physical Analysis of GPCRs

Structure-based, rational drug discovery is widely employed by the pharmaceutical industry to design compounds with optimal specificity and potency to molecular targets, often at soluble enzymes such as protein kinases. The structural information provides information on the physical properties of the ligand binding site(s) and helps to design pharmacophore models of the ligand when docked. This provides the foundation for screening structural families of molecules with predictable chemical characteristics as well as optimal functional properties to modulate the molecular target [35].

This approach has not been used widely in the GPCR field, because GPCRs are not easily crystalized for structural evaluation. However, within the last decade, six different GPCRs have been crystalized in their inactive form, including the avian β_1_- adrenoceptor [8], human β_2_-adrenoceptor [4-6], human adenosine A_2A_ receptor [7], human dopamine D3 receptor [9], human CXCR4 receptor [10], as well as rhodopsin [11]. The structural data from these studies not only provides critical information on the molecular nature of drug interaction with GPCRs, but also provides approaches that allow for crystallization of other GPCRs, revealing a more rational method for drug development.

Rhodopsin was the first full receptor to be subjected to X-ray analysis [11,36-40]. However, the analysis of more ‘druggable’ GPCRs only occurred with the development of approaches to grow the entire β-adrenoceptor as crystals [3,4,41]. GPCRs are difficult to grow as crystals because they are structurally unstable in purified form. Kobilka and Deupi [3] attributed this instability to the unordered structure of the large third intracellular loops of the receptors. To overcome this problem, Rosenbaum *et al.* [4] replaced the large third intracellular loop of the β_2_-adrenoceptor with T4 lysozyme, a sequence that is more structurally rigid and polar than the native intracellular loop of the receptor. This conferred greater stability on the receptor as a whole and had the advantage that the mutant receptor could be expressed at high levels in a crystal form for X-ray spectroscopy. T4 lysozyme was likewise inserted into the CXCR4 and dopamine D3 receptors to generate crystals [9,10]. For studies on the β_1_-adrenoceptor, Warne *et al.* [8] truncated the termini of the receptor to reduce proteolytic cleavage and made mutations of the receptor that increased thermal stability to allow for crystallization as was also done on the D3 receptor [9]. In all of these cases, to generate GPCR crystals, the functional moieties of the receptor needed for G protein coupling were altered to form a protein sufficiently stable for the rigorous physical procedures needed for crystallization. These mutant receptors could be analyzed by radioligand binding and possessed expected affinities for antagonists and inverse agonists. As the receptors were unable to couple to G proteins, to establish functionality Rosenbaum *et al.* [4] inserted flurophores in cysteine residues in transmembrane 6 of the receptor. These allowed detection of conformational changes induced by ligand binding as a measure of activity of the mutant receptor. They found that the full agonist, isoproterenol, produced a greater change in fluorescent intensity than the partial agonist, salbutamol while inverse agonists induced no or little conformational shift on the β_2_-adrenoceptor.

These authors then conducted structural analysis of the mutant receptor bound to the high affinity inverse agonist, carazolol which further stabilized the receptor. This allowed for analysis of the physical sites and amino acids critical for ligand binding particularly in the hydrophobic pore of the receptor. Similar studies have now been done on other druggable GPCRs. These studies revealed common structural requirements in ligand binding to the orthosteric domain in the transmembrane core. Such information, along with the extensive physical data on these and other GPCRs is now being used to develop computational models of the nature of small molecules binding to GPCRs, thus facilitating medicinal chemistry efforts to discover novel drugs.

To this point, Kolb *et al.* [42] used crystal structure information of the β-adrenoceptor as a basis for virtual screening of a chemical library of a million compounds. They employed a molecular docking program (DOCK3.5.54) for their studies, which assesses compounds for interaction at a binding site on the receptor. From the virtual screen, 25 compounds were selected and then tested functionally on the receptor. Some bound with nanomolar affinities and six were identified as potent inverse agonists. Since the crystal structure analysis is biased towards evaluation of carazolol binding, this finding was not unexpected. Similarly, Michino *et al.* [14] have recently described a docking model based on physical analysis of the adenosine A2 receptor referred to as “GPCR DOCK 2008” which can be employed for similar virtual screening. Presumably, as more extensive structural information arises on the GPCRs, these analyses will be further refined and possibly broadened to facilitate binding of other ligands such as agonists.

In fact, Ivetac and McCammon [13] have recently mapped potential allosteric binding sites on the β_1_- and β_2_-adrenoceptors using the structural information from the crystallographic studies and the FTMAP algorithm employing molecular dynamic modeling. Essentially, the computational analysis identifies binding pockets on the receptor surface that could constitute allosteric sites for ligand interaction. Similarly, using the crystal structure analysis of rhodopsin, Taylor *et al.* [45] employed virtual screening using a peptidomimetic structure which experimentally had been shown to stabilize the rhodopsin-G protein state. A pharmacophore model was developed from the peptidomimetic to screen scaffolds of commercial and publicly available small molecule libraries. Three of the compounds found to bind to rhodopsin in experimental studies interacted with allosteric sites, suggesting that these computational approaches can be employed in a rational approach to identify allosteric compounds binding outside the orthosteric core of GPCRs.

The studies on the crystal structure analysis of the mutant GPCRs provides important information on the static structure of ligand binding to GPCRs. However, the information gained has its limits, in particular with regards to how agonists cause activation. While Rosenbaum *et al.* [4] actually reported that agonists bound to the mutant β_2_-adrenoceptor with high affinity, they attributed this to the formation of a constitutively active nature of the receptor conferred by the mutations. The absence of the G protein binding domains of the receptor, a critical allosteric modulator of agonist binding, puts limits on the utility of the structural analysis of agonist binding for rational drug design. However, crystal structure analysis is not the only physical method used to study GPCRs. In fact, to better investigate the conformational changes induced in the wild-type β_2_-adrenoceptor by agonist binding, Bokoch *et al.* [43] for the first time employed NMR spectroscopy on a full length druggable GPCR. At around the same time, nuclear magnetic resonance (NMR) analysis of rhodopsin was also described by Gautier *et al.* [44]. Unlike crystal structure analysis, NMR can provide greater insight into protein dynamics of ligand binding and is extensively used to provide critical information for rational drug design against other molecular targets such as kinases.

Bokoch *et al.* [43] focused their studies on investigating dynamic changes occurring around a natural salt bridge formed between extracellular loops 2 and 3 of the β-adrenoceptor that make up the extracellular limits of the transmembrane core of the orthosteric binding domain. The authors reasoned that agonist induced conformational changes would be expected in this region based on the crystal structure analysis of carazolol binding site as well as from fluorescent spectroscopy studies done in the past by this group on this receptor in studies on agonist binding. Importantly, they speculated that agonists would likely either break the salt bridge or alter the conformation around this region of the receptor. Furthermore, they suggested that studies on the surface dynamics of the receptor using NMR would be important in understanding the role of extracellular loops of the receptor in mediating conformational changes needed for receptor activation, and that those regions might be fertile sites for allosteric ligand binding and regulation.

In fact, they noted that extracellular loops may be important sites for discovery of better and more efficient receptor subtype selective drugs. For example, the orthosteric binding domains of the β_1_- and β_2_-adrenoceptors are almost identical with 15 or 16 amino acids being the same. In contrast, the extracellular loops of the receptor differ greatly. If the loops are critical in transducing orthosteric agonist induced conformational changes, then small molecules binding to those allosteric sites might be expected to either serve to block or enhance agonism without directly affecting agonist binding and since the loops differ substantially in sequence, then drugs targeting those sites may have greatly enhanced subtype selectivity.

Bokoch *et al.* [43] did NMR spectrum analysis of the β_2_-adrenoceptor by labeling a lysine group near the salt bridge with ^13^C-methyl group which served as a probe for the NMR analysis. This modification did not alter the crystal structure of the receptor nor did it affect ligand binding or G protein coupling of the wild-type receptor. Crystal structure analysis showed that the inverse agonist carazolol interacted with a phenylalanine (Phe) at position 193 in the receptor, adjacent to the start of the salt bridge of the receptor and carazolol produced a shift in conformation of the second and third extracellular loops. Other inverse agonists produced similar shifts and the authors speculated the conformational shifts were due to the tricyclic or bicyclic aromatic rings of the inverse agonists interacting with Phe^193^. In contrast, the neutral antagonist alprenolol, which has only a single aromatic ring, did not cause a conformational shift because this molecule is unable to effectively interact with Phe^193^ in the carazolol binding pocket. This information provides structural clarity on the nature of differences in neutral antagonist and inverse agonist binding to this receptor.

Agonist binding also induced conformational changes in the receptor that differed considerably from inverse agonist binding. The agonist binding causes a shift in the structure of the salt bridge connecting extracellular loops 2 and 3, focused on lysine (Lys) 305, the other end of the bridge from the Phe^193^. Agonists appear to cause movement of the two extracellular loops. In contrast, inverse agonists appear to stabilize the salt bridge at Phe^193^ to reduce movement of the extracellular loops. Thus, in the model developed, the unbound GPCR in a basal state exerts flexibility in its third intracellular loop and extracellular loops, and can couple to G protein to produce basal GTPase activity. Inverse agonists stabilize the salt bridge to prevent basal conformational shifting of the receptor to uncouple from G protein to inhibit basal GTPase activity. Agonists cause profound movement of the extracellular loops to orientate the receptor to a most favorable conformation to couple to G protein to increase G protein coupling.

Most importantly, the authors [43] speculated that ligands binding to the extracellular surface of the receptor, distinct from the orthosteric binding pore but within regions of extracellular loops 2 and 3 could modulate the conformational shifts induced by agonists, either potentiating (allosteric activator) or by blocking (allosteric inhibitor). This suggestion is consistent with data from modeling studies by Ivetac and McCammon [13] who showed potential binding pockets at the mouth of the orthosteric binding pore of the β-adrenoceptor. They indicated that similar allosteric sites have been identified in muscarinic receptors, and ligand binding to these pockets could act by blocking ability of orthosteric ligands from gaining entrance to the orthosteric pore, acting as a sort of gate keeper. In effect, allosteric inhibitors could stabilize a “closed” form of the GPCR preventing access of orthosteric agonists.

The model however does not explain how allosteric activators would work. Consistent with the NMR analysis of Bokoch *et al.* [43], such activators could bind to regions near extracellular loops 2 and 3 to facilitate conformational shifts induced by agonists to enhance G protein coupling and could also explain the actions of dualisteric ligands, which are able to bind within the orthosteric pore to activate receptor and also interact with surface regions of the receptor to tweak conformational changes needed for receptor stimulation. While the authors acknowledge that they have limited information on the generality of their data to other GPCRs, the data provides a useful model to begin to design allosteric modulators of GPCRs and implies that NMR could be used to directly test efficacy of such compounds.

The focus of most of the crystal structure and NMR analysis has been on receptors which bind endogenous ligands which are small molecules, such as dopamine, epinephrine, norepinephrine and adenosine. The models and information gained from these studies show how ligands interact with a central hydrophobic core of the GPCRs. However, a large number of GPCRs have large endogenous ligands and those molecules do not easily fit into a hydrophobic binding pocket first described for β-adrenoceptor. In fact, it has been shown that for peptide receptors, such as the opioid receptors, the endogenous ligands, including enkephalins, endorphins and dynorphins appear to primarily bind to orthosteric domains in the extracellular loops of the receptors [46-49]. For example, dynorphin A, the endogenous ligand for the kappa opioid receptor has been shown by mutagenesis studies, NMR analysis and molecular modeling to bind to the second extracellular loop of the kappa receptor [49-54]. In contrast, small molecule selective antagonists bind to different regions [46,57]. For other opiate receptors, the N-terminus and extracellular loops 1 and 3 appear to be critical regions involved in selective peptide agonist [55,56,58,59-61].

Importantly, the recent crystal structure analysis of the CXCR4 receptor bound to a small molecule antagonist and a cyclic peptide show the ligands bind to pockets more closely associated with the extracellular domains of this GPCR [10]. The endogenous ligands for CXCR4 receptors are chemokines which are small proteins which would not be expected to easily fit into a deep hydrophobic pocket as found with the adrenoceptors. The structural studies support the notion that differences exist in the manner by which peptide or protein endogenous ligands bind to GPCRs compared to how endogenous small molecule ligands bind to their receptors.

In fact, NMR has been employed to study the binding of a number of different endogenous peptides and their agonists to fragments of GPCRs (not the entire receptor) [53,54,62]. For example, CCK8 was shown to bind to the N-terminus and extracellular loop 3 of the CCK1 and CCK2 receptors by NMR analysis [63,64] which provided information on the specific amino acids in these extracellular domains that the ligand makes contact with and the helical nature and structures conferred on the receptor upon binding. For these receptors it was also found that non-peptide agonists had overlapping binding domains in extracellular loop 3 with CCK8 [65].

Similarly, for Class B1 GPCRs which include the peptide receptors for corticotropin releasing factor (CRF), glucagon-like peptide-1 (GLP-1), gastric inhibitory peptide (GIP), pituitary adenylate cyclase activating peptide (PACAP), parathyroid hormone (PTH) and calcitonin gene-related peptide (CGRP), both crystal structure analysis and NMR studies on fragments of the receptors have shown that both endogenous ligands and peptide antagonists bind to the N-termini of the receptors [66-71]. In the case of CRF, binding to the N-terminus of the CRF1 receptor induces helical formation of the peptide ligand and binding to the N-terminus of the receptor occurs along the hydrophobic face of the ligand [67,68]. The binding of CRF to the N-terminus induces a conformational change in the receptor to orientate the N-terminal segment of the ligand to make contact with the extracellular loops of the receptor needed for receptor activation. Thus, these physical studies clearly show that binding of endogenous ligands to peptide receptors have different orthosteric binding domains than small molecule hormones binding to GPCRs such as the β-adrenoceptor.

This however, does not negate the important nature of the crystal structure and NMR information on the β-adrenoceptor nor its application in understanding how GPCRs work in general. In fact, the studies by Bokoch *et al.* [43] and Ivetac and McCammon [13], suggest that multiple binding sites, both in the hydrophobic core and the extracellular loops can influence both activation and inhibition of GPCRs. Thus, while sites in the extracellular loops of the β-adrenoceptor may provide targets for allosteric modulation and activation, sites in the extracellular loops of peptide receptors may serve as primary sites of orthosteric ligand binding. These extracellular sites can provide a rich source of targets for new small molecule drug discovery at either set of receptors. Thus, physical analysis and modeling based on the β-adrenoceptor is likely to be extremely useful in development of novel ligands and drugs acting upon peptide receptors and GPCRs in general.

## 3. Allosteric Modulators

Most drugs developed against GPCRs bind to the orthosteric ligand domain. However, as described above, GPCRs have many other extracellular facets besides their orthosteric binding domains that can affect receptor activity and function that can serve as drug binding targets [22-28]. Furthermore, intracellular domains of GPCRs couple to G proteins and interact with a variety of protein kinases and the β-arrestin family [72]. These intracellular sites are physically distinct from the orthosteric binding domain and can bind allosteric modulators. Small molecules that bind to allosteric regions of GPCRs have properties distinct from classical orthosteric ligands and can provide potential therapeutic advantages over classical GPCR drugs [1,2,22-24]. There are at least five unique properties of allosteric modulators. First, they can be highly selective for individual GPCRs because the structures of their binding pockets are unique for each receptor. For example, orthosteric binding domains of subtypes of receptors for some neurotransmitters, such as serotonin, dopamine and acetylcholine, can have similar affinities for the endogenous ligand. For this reason, it can be difficult to develop subtype selective drugs targeting the receptor subtypes because the subtypes have similar ligand binding proteins. However, allosteric sites on receptor subtypes do not generally have endogenous ligands. As a consequence, the determinants for their binding sites may be different amongst receptor subtypes providing approaches to more easily develop subtype selective ligands.

Similarly, some GPCR subfamilies have unique properties that make the discovery of orthostatic agonists or antagonists difficult to accomplish. An example is the protease activated receptor (PAR) subfamily which mediates the biological functions of thrombin. Thrombin is a protease and a major physiological factor in blood coagulation and wound healing. Thrombin activates PAR through a unique mechanism in which the protease cleaves the N-terminus of the receptor and the resulting truncated receptor bends back upon the receptor to serve as a tethered ligand to cause continuous activation. Efforts to develop anti-thrombin drugs have primarily focused on inhibiting thrombin activity or in some cases generating PAR receptor orthostatic binding antagonists. Because of the unique manner in which thrombin binds to PAR, development of small molecule antagonists and agonists has proven difficult. However, allosteric enhancers or inhibitors need not bind to PAR in the same manner as thrombin and therefore are not constrained in their receptor interaction as orthostatic agonists or antagonists. As described below, allosteric PAR modulators are beginning to be developed that provide a unique family of drugs targeting this clinically important GPCR family.

Secondly, allosteric ligands do not compete with endogenous transmitters or hormones like orthosteric ligands. As a consequence, allosteric modulators generally can be employed at lower dosing than orthosteric ligands to be effective. Thirdly, allosteric modulators are less likely to desensitize like orthosteric agonists. For allosteric activators, this property means they might be less prone to tolerance development, which plagues many orthosteric agonists, such as in the case of opiate analgesics. Fourthly, allosteric modulators tend to only effect the agonist occupied GPCR without causing actions of their own, reducing toxicity potential. As a consequence, allosteric modulators are usually found to have a ceiling effect, since when the receptor is fully occupied by an orthosteric agonist, the allosteric modulator can produce no further effect, no matter what concentration is employed. This allows allosteric modulators to temper activity of receptors rather than simply turning them off or on, providing a means to fine-tune GPCR activity.

Finally, allosteric modulators have the capacity to affect some functions of an individual GPCR and not others. In fact, as described by Rajagopal *et al.* [72] there are already examples of orthosteric agonists producing biased agonism by directing the receptor to selective G protein activation or distinct signaling pathways such as the β-arrestin pathway. For example, most muscarinic agonists can activate both G_s_ and G_q_ signaling pathways. However, some muscarinic orthosteric agonists only stimulate G_q_ signaling [72,73]. Similar differentiation of signaling pathway activation is found with angiotensin receptors where some ligands activate G proteins while others, acting through the same receptor, only stimulate β-arrestin signaling pathways involving mitogen-activated protein kinases (MAPKs), SRC, nuclear factor-κB (NF-κB) and phosphoinositide 3-kinase (PI3K). Thus, bias agonism can come about when the receptor is directed to couple with some but not all of their signaling pathways through different conformational changes.

In a similar manner, allosteric modulators can block or enhance GPCR coupling to one G protein and not affect coupling to other G proteins or signaling pathways. Furthermore, allosteric modulators that can prevent β-adrenoceptor kinase (βARK) and β-arrestin interaction with GPCRs may prevent desensitization, thereby providing an approach to prolong the activity of orthosteric agonists.

One of the most obvious examples of allosterism that focuses on G protein coupling domains of GPCRs is found with pepducins. These are ligands corresponding to specific sequences of the intracellular domain of a particular receptor designed to either block receptor coupling or mimic receptor coupling to a particular G protein to produce desired cellular effects. The compounds are synthesized with membrane anchoring segments which allow the compound to enter cells and localize to the cell membrane to be in close proximity to the GPCR target. Because the intracellular sequences of the GPCRs can differ, the pepducins can have high GPCR selectivity as well as target selective functions of a receptor.

Thus, allosteric ligands provide greater diversity in selecting desired functional and potentially clinical outcomes than orthostatic agonists and antagonists. Specially, with respect to orthostatic ligands, allosteric modulators provide greater specificity in targeting individual GPCRs, and can temper receptor activation in a manner not found with agonists. Furthermore, they can direct the activation of individual GPCRs to mediate some but not all of the functions of endogenous ligands and agonists at a given receptor by causing preferred GPCR-G protein interactions. Importantly, structural information now exists for some allosteric sites to allow for rational drug design and high throughput screening (HTS) assays have been developed to identify allosteric modulators. Consequently, the tools needed to begin to develop families of allosteric modulators for targeted GPCRs are at hand to generate new allosteric therapeutics.

### 3.1. Examples of Allosteric Modulators

Cinacalcet (Sensipar™) was the first allosteric GPCR modulator to gain FDA marketing approval. This was followed by Maraviroc (Celzentry™). These drugs were identified using conventional drug screening technologies and only later on were found to act in an allosteric manner to affect GPCR function.

Cinacalcet is a positive allosteric modulator of the Ca^++^ sensing receptor (CaR). This receptor is a member of the type C family of GPCRs, characterized by large N-terminal regions. Ca^++^ binds to the N-terminus and a critical role of this receptor is to regulate circulating Ca^++^ levels to maintain control of parathyroid hormone (PTH) release and levels. Other endogenous ligands at this receptor are Mg^++^ and amino acids which also bind to the N-terminus. In contrast to Ca^++^ and the other orthosteric ligands, cinacalcet is a small molecule that binds to the transmembrane region of the receptor to alter conformation to enhance affinity for Ca^++^ [74].

The compound is therapeutically useful because in kidney disease, hypocalcinemia results in excessive PTH which causes bone fractures, pain and cardiovascular disorders. Cinacalcet increases sensitivity of CaR to Ca^++^ to suppress excess PTH release despite hypocalcemia. The drug does not appear to have intrinsic activity and its actions are dependent on the binding of Ca^++^ to the receptor. Thus, cinacalcet is an allosteric modulator that binds to the transmembrane regions and hydrophobic pocket of the CaR distal to the orthosteric binding domains in the N-terminus.

Maraviroc has received marketing approval from the FDA for treating HIV disease [75,76]. It is an allosteric inhibitor of the CCR5 receptor. Maraviroc was shown to be an allosteric inhibitor by its ability to differentially affect the binding of different chemokines to the receptors. Similarly, the CCR5 allosteric inhibitor aplaviroc was shown to block the binding of the chemokine agonist CCL3 to the receptor but not the chemokine agonist CCL5 [77]. Orthosteric antagonists should block all chemokine binding to the receptor [78].

Maraviroc's therapeutic mechanism of action, like a number of other allosteric CCR5 inhibitors, is believed to involve preventing HIV entry into immune cells via the CCR5 receptor. Interestingly, maraviroc is more potent in blocking HIV entry than in affecting chemokine induced internalization of the receptor. This is important, because chemokines are known to be useful in treating HIV disease, in part because they can cause internalization of the CCR5 receptor removing the conduit for HIV entry. Thus, the allosteric inhibitor maraviroc induces conformational changes in CCR5 receptor to block interaction of HIV and also allows native chemokine internalization of the receptor, further preventing HIV infection [78]. Such activity would be difficult for a classical orthosteric antagonist to accomplish.

Another allosteric activator with potential clinical utility as an antipsychotic is the muscarinic M4 receptor modulator LY2033298. There are five subtypes of muscarinic receptors and as with the biogenic amine receptors, it has been difficult to identify subtype selective agonists because of similarities of the orthosteric binding pocket. In an effort to identify allosteric activators selective for the individual receptors, the pharmaceutical company Lilly and it's collaborators [79] screened the five cloned human receptor subtypes in calcium assays both in the absence of activating ligand and in the presence of a submaximal concentration of acetylcholine. The premise of this assay is that allosteric activators would be expected to produce no or limited agonism by themselves but would be predicted to potentiate activation by orthosteric agonists. This is in fact how LY2033298 was identified as an M4 selective allosteric modulator. LY2033298 was also found to potentiate agonist binding to the receptor but had minimal effect on antagonist binding reinforcing the allosteric nature of the ligand.

Furthermore, mutagenesis studies indicated that LY2033298 interacts with determinants in the third extracellular loop of the M4 receptor [79]. Later studies identified additional sites in the first and second extracellular loops needed for LY2033298 actions [80]. In contrast, orthosteric agonists and antagonists bind to transmembrane regions making up the hydrophobic core pocket of the receptor. This finding is of interest in the context of the results of Bokoch *et al.* [43] who suggested that ligands binding to the extracellular loops of the β-adrenoceptor may have allosteric properties by affecting the ability of orthosteric agonists to induce conformational movements of the extracellular loops. In fact, many of the structural requirements of muscarinic and adrenoceptor ligand requirements are similar suggesting the information described above in identify allosteric sites of GPCRs and employing rational drug design for these sites may hold true for muscarinic receptors and likely class A small molecule GPCRs in general.

LY2033298 acts *in vivo* as an allosteric enhancer. On several cholinergic behaviors, including conditioned avoidance responding and prepulse inhibition, it produced limited or no effect. However, it greatly potentiated the actions of the cholinergic agonist oxotremorine on these behavioral paradigms. These studies and others suggest that LY2033298 as well as several other M4 allosteric activators have preclinical efficacy as anti-psychotics and may have utility as a new class of drugs to treat Schizophrenia, with the added advantage that the drugs produce no or limited actions on their own.

A number of potentially therapeutically useful allosteric modulators have been discovered against the metabotrophic glutamate receptors (mGluR). These receptors are members of the type C family of GPCRs, like the CaR described above which have large N-termini. Glutamate binds to these receptors in the N-terminal regions in a structure referred to as the venus fly trap domain [81] and binding induces conformational changes transmitted to the transmembrane domains to affect G protein coupling and signaling.

The mGluRs have prominent role in CNS physiology given that glutamate is one of the major transmitters in brain. Furthermore, abundant information is available that the receptor subtypes may mediate distinct functions of glutamate in brain. However, the mGluRs, like many other receptors have suffered from the lack of subtype selective drugs, which is why developing allosteric modulators is an attractive idea to develop drugs targeting each receptor subtype. Because the orthosteric binding domain of the receptors is in the N-terminus, one approach employed to discover allosteric modulators is to use N-terminal truncated receptors or chimeric receptors to screen for compounds binding to the transmembrane region to affect signaling [82-84].

The truncated mGluRs have been employed to identify the binding regions of one of the most well studied allosteric inhibitor of mGluR5, MPEP [84,85]. mGluR5 has been speculated to have a role in a number of behaviors and disorders, including anxiety, autism, Fragile X, pain and neurodegenerative disorders such as Parkinson's and Huntington's diseases. MPEP was one of the first compounds able to selectively block mGluR5 and consequently was used to study the behaviors linked to this receptor subtype. In fact, another allosteric inhibitor of mGluR5, fenobam has been tested in clinical trials in humans and been found to be safe. Initial studies have shown some efficacy in treating anxiety. This is particularly important because of the side effect profile of available anti-anxiety agents, such as the benzodiazepines. Fenobam has also been tested for treating fragile X in humans and shown effective, at least in initial studies [86]. Fenobam is now being tested clinically for treating a number of disorders including L-dopa induced dyskensias in Parkinson's disease and in Huntington's disease (see clintrials.gov). Similarly, because of the high selectivity, other mGluR5 allosteric inhibitors are being tested clinically in PET studies as diagnostic markers for a number of different diseases (see clintrials.gov).

### 3.2. Dualsteric Ligands

While the classic concept of allosterism focuses on compounds that bind to sites physically distinct from the orthosteric binding domain, ligands are now being designed to bridge the gap to bind to the orthosteric and allosteric sites simultaneously. These so called dualsteric or bitopic molecules can provide several advantages over simple orthosteric or allosteric ligands. First, they can be highly GPCR selective, due to the requirement to bind to both the orthosteric and allosteric sites. Secondly, much like biased agonists, they can be designed to affect some signaling pathways and not others.

Antony *et al.* [25] described some of the first dualsteric compounds against muscarinic receptors. They synthesized a compound containing the orthosteric agonist oxotemorine with bis(ammonio)-alkane-type allosteric fragments selective for the M2 muscarinic receptor. Oxotremorine is not selective for any muscarinic receptor subtype, but the bis(ammonio)alkane-type moiety was highly selective for the M2 receptor. To test whether the dualsteric compounds bound to the orthosteric and allosteric sites in the receptor, they generated point mutations in the receptor known to either prevent binding of orthosteric ligands or allosteric ligands. Each mutation reduced affinity of the fused ligand in a manner consistent with loss of either binding to orthosteric or allosteric sites. The dualsteric ligands stimulated receptor-G protein coupling via interaction with the orthosteric site and efficacy was blocked by orthosteric antagonists (atropine) but not by allosteric inhibitors (W84).

They then measured functional responses of the dualsteric ligand using dynamic mass redistribution (DMR). DMR measures movement of the mass within cell in a population as a response to drug action [87-89]. The concept is that drugs that act on cells can affect signaling pathways which can lead to translocation of proteins and molecules from one site in a cell to another and this redistribution can be detected as a change in refraction of light beamed through the cells using biosensors, such as the Corning Epic system. Very small changes in mass just under the surface of the cell membrane bound to the biosensor surface change the refractive index and consequently the wavelength of the reflected light which is detected by the Epic reader as the phenotypic response to a drugs action on the cells.

A number of studies [87-89] have employed DMR to measure responses of GPCRs including endogenous GPCRs in cell lines and found that the DMR responses were consistent with the known pharmacological specificity's of those receptors. Furthermore, they were able to identify DMR signature responses based on the G protein subtype associated with the receptor. That is, DMR responses of G_s_, G_q_ and G_i_ coupled receptors differed from each other but different receptor linked to the same G protein gave similar types of responses.

Antony *et al.* [25] showed that in CHO cells expressing the human muscarinic receptors, orthosteric agonists stimulated both G_i/o_ and G_s_ pathways and produced DMR responses similar to those induced by the dualsteric ligands. However, in cells treated with pertussis toxin to inactivate G_i/o_, orthosteric agonists induced a DMR response but dualsteric ligands did not, suggesting the dualsteric agonist directed the M2 receptor to couple selectively to a G_i/o_ signaling pathway.

Interestingly, the authors then tested the dualsteric ligands in tissues that differentially express native muscarinic receptor subtypes, including guinea pig left atrium, which predominantly expressed M2 receptors, rabbit vas deferens which mainly expresses M1 receptors and possibly M4 receptors and guinea pig ileum, which expresses M2 and M3 receptors. Because the orthosteric component of the ligand is an oxotremorine analog, the dualsteric ligands maximally stimulated all three tissues compared to non-selective orthosteric agonist. However, the potencies of the dualsteric ligands for stimulating the left atrial tissue where much greater than for the other tissues, indicative of the allosteric component primarily affecting M2 receptor affinity for the ligand. Thus, the dualsteric ligand not only makes for highly selective M2 agonist, but directed the agonism to selectively affect one signaling pathway over another, in contrast to the more promiscuous orthosteric agonists.

### 3.3. Allosteric Modulators of GPCR-G Protein Coupling: Pepducins

The intracellular domains of GPCRs are key sites for allosteric regulation by G proteins and other regulatory proteins such as kinases, including βARK and β-arrestins. While agonist binding to GPCRs induces conformational changes to cause G protein activation, the interaction of G proteins with receptors also induces conformational changes to affect affinity of the receptor for agonists. There are multiple regions in the cytoplasmic domains of GPCRs to which G proteins can make contact, with the polar intracellular loops and the C-terminal tail being the ones most studied. These regions have also been the target for development of allosteric compounds that can affect orthosteric ligand binding but also, most importantly, GPCR signaling. Since GPCRs are capable of coupling to more than one G protein, and able to affect multiple signaling pathways in cells, allosteric modulators targeting these intracellular domains have the ability to select for functions of the receptors.

There are at least two approaches that have been used to develop allosteric modulators of GPCRs targeting intracellular G protein coupling domains. In one, developed by Gilchrist and colleagues [22,90,91], C-terminal peptide fragments of G proteins were used to bind to GPCRs to identify receptor/G protein coupling domains. This approach was based on the premise that the G_α_ subunits interact with their cognate GPCRs via their C-terminal region. The C-terminal peptide fragments were capable of blocking GPCR signaling. This was shown for G_αt_ activation by rhodopsin, G_αs_ activation by β-adrenoceptors, and blockade of G protein activation by the CXCR1, PAR1 and dopamine D5 receptor stimulation. Furthermore, these G protein peptide fragments were selective in blocking binding of GPCRs to some G proteins but not others. Using this approach, Gilchrist and associates developed a high throughput screening (HTS) assay for small molecules that affect interaction of GPCRs with G_α_ peptide fragments to identify potential allosteric modulators.

A second approach to identify drugs targeting the G protein coupling domains of GPCRs has been developed by Anchor Therapeutics and their associates and employs molecules referred to as pepducins as GPCR drugs [92-96]. These molecules, which were described previously above, are peptide fragments of the intracellular loops of GPCRs which are synthesized with N-terminal lipid tails. The charges in the peptide are neutralized and the lipid moiety allows the pepducin to enter cells and the tail anchors the peptide fragment near the cell membrane, concentrating the pepducin to where the GPCRs are located. Biophysical studies using fluorescent measurements have shown pepducin insertion and internalization into cells and functional studies have shown that they can selectively affect GPCR function. Pepducins were developed that could act as allosteric activators or inhibitors of the PAR family as well as CXCR1, CXCR2 and CXCR4 [92-96].

Despite the concerns of using peptides as drugs, pepducins have also been shown to be effective *in vivo* in animal studies. For example, pepducins targeting PAR4 could prevent platelet activation and prolonged bleeding, consistent with antagonism of PAR4 function [92,95-98]. Similarly, PAR1 pepducins blocked thrombosis in guinea pigs and prevented clotting of human blood *in vitro* [99] and blocked sepsis-induced vascular damage *in vivo* [100].

PAR1 pepducins were also effective in preclinical studies on breast cancer. While PAR1 is not expressed in the epithelia of normal breast tissue, it is highly expressed in carcinomas. Yang *et al.* [101] showed that PAR1 pepducin inhibitors killed breast cancer cells expressing PAR1 *in vitro* and induced death of breast carcinoma xenografts and prevented metastasis. The actions of the pepducins were dependent on the cellular expression of the receptor to which they were targeted. Similar results were found with PAR1 pepducins in preclinical studies to treat ovarian cancer [102]. These studies indicate pepducins could have utility in a variety of inflammatory and proliferative disorders.

The utility of pepducins targeting PAR1 and PAR4 is particularly important, since despite the clinical importance of these receptors [96-99], they are notoriously difficult to develop small molecule antagonists. No PAR1 antagonist has received FDA approval and only a few have reached clinical development. This may be due to the unique nature of the activation of the PAR1 receptor where thrombin binds to and cleaves the N-terminal region of the receptor and the resultant truncated portion of the receptor acts as a tethered ligand, binding to extracellular loop 2 to cause activation of the receptor. As a consequence, agonists or antagonists targeted to the orthostatic binding domain of the receptors are difficult to develop. Agonists would somehow have to either act like thrombin to cleave the receptor or induce conformational changes in the N-terminus so that this region acts in a similar manner as a tethered ligand. Orthostatic antagonists would have to bind to the thrombin cleavage site to prevent access of thrombin. Generally, small molecule inhibitors of protein-protein (thrombin and the receptor) interaction are not easily identified.

In contrast, pepducins would not have to deal with the unusual nature of thrombin activation of the PARs. Instead, they would target the intracellular domains of the PARs involved in G protein coupling and receptor signaling. Thus, pepducins not only may be useful in developing novel PAR1 therapeutics but are likely to be useful in developing probes and drugs against a number of recalcitrant receptors for which orthosteric drug discovery has generally been difficult.

In fact, an important aspect about pepducins is that rational design can be employed in their development. Much information is available on the structure of the intracellular loops of GPCRs including both crystal structure analysis and NMR data [9,37,38,103-105]. Furthermore, modeling studies have already identified regions of allosterism in these domains which can be employed for selection of peptide fragments most likely to affect GPCR-G protein coupling. Importantly, since the intracellular domains have significant sequence divergence amongst the GPCRs, there is significant likelihood of identifying pepducins highly selective for individual receptors.

## 4. Oligomerization of GPCRs

Classically, GPCRs have been viewed as monomeric proteins. However, evidence has accumulated that GPCRs may physically interact with each other and that oligomeric forms of the same receptor (homodimers) or different receptors (heterodimers) may be functionally active [29-34,106,107]. The evidence for oligomerization has been based on biochemical co-immunoprecipitation studies using antibodies directed at one or the other dimer component [107]. This has involved studies using epitope tagged receptors transfected into cells and using antibodies against the tag on one receptor to co-immunoprecipitate the other receptor. Antibodies against the receptors themselves have also been employed to co-immunoprecipitate receptor heterodimers. Furthermore, biophysical approaches have also been used to measure dimer formation. These studies have employed fluorescence resonance energy transfer (FRET), time resolved FRET (TR-FRET), fluorescence recovery after photobleaching (FRAP), internal reflection fluorescence microscopy (TIRFM) and bioluminescence resonance energy transfer (BRET) based assays to measure either homodimers or heterodimers association [32,33,108-112]. Furthermore, complementation assays have been employed to measure oligomerization in which two receptors are tagged on their N- or C-termini with different constructs, each by themselves unable to give off a fluorescent response but when in close proximity to each other, as in a dimer, form a complement that gives off a luminescent response [113-115]. Finally, studies by Wu *et al.* [10] showed in crystal structure analysis that homodimers of the CXCR4 receptor form and these authors described the physical nature of this interaction and provided information not only on the sites of contact of the receptor monomers but an explanation of how the nature of the contact could explain the promiscuity of this receptor to form dimers with other GPCRs both within its receptor subfamily and with other receptor types.

Pharmacological approaches have also been employed to establish dimer formation. Studies by Zheng *et al.* [116] studied the interaction of mu opioid receptors with CCK2 receptors. These receptors are co-expressed in brain regions involved in pain modulation and a number of studies had suggested that the mu opioid and CCK systems may interact and that CCK antagonist may potentiate morphine induced analgesia and diminish mu receptor mediated tolerance development.

These authors investigated whether oligomer formation may be a basis of the interaction of the mu opioid and CCK system. They used a BRET assay to measure interaction of the cloned mu opioid and CCK2 receptors. Under basal conditions the receptors did not appear to associate and mu agonist and CCK ligands did not induce oligomerization as measured in the BRET assay. However, bivalent ligands containing a mu agonist and a CCK2 antagonist did cause a BRET response, suggesting that they bound simultaneously to each component of the dimer to promote oligomerization. Similar results were reported by Harikumar *et al.* [117] in which a fluorescent complement assay was employed to measure dimer formation and in these studies, the authors were able to fine tune the linker size between the opioid and CCK pharmacophore to measures distances of the ligand parts needed to promote dimer formation. Thus, these studies indicate that dimer formation can be induced by single ligands designed to bind simultaneously to two different receptors and suggest an approach to designing novel drugs based on their selective interaction with oligomers.

Some studies indicate that dimers may have pharmacological properties that differ from the monomers. Most studies on the pharmacological properties of dimers have shown that the pharmacological profile of ligands binding to monomers and oligomers are similar but that the affinities of the ligands may differ [34,118]. However, there are examples of compounds being identified that either bind to the oligomer differently or produce agonism or antagonism at the oligomer but not at the individual monomeric receptors [34,119,120]. These findings suggest that it is possible to identify small molecules that may selectively target dimers. However, as discussed by Birdsall [118], caution is needed in interpreting the pharmacological studies that suggest dimers exhibit differences in ligand binding properties from monomers. As suggested by Birdsall [118], in many cases, ligand binding characteristics “…do not agree with the thermodynamic constraints…” of the available models describing dimer interaction.

In fact, determining whether dimers, especially heterodimers, have pharmacologies that can be distinguished from monomers of each receptor could be more clearly established if compounds, such as the two examples indicated above [119,120] are identified that bind to or affect the activity of the heterodimer but not the homodimer or monomer. Identifying such compounds would support that notion that the ligand binding pocket of the heterodimer is different from the homodimer or monomer and the physical determinants of the heterodimer are unique.

This may be possible in the future since assays exist that can be employed to select for drugs targeting dimers. Most of the luminescent assays to measure dimer formation, in which acceptor and donors are attached to different components of the oligomer, can be employed to identify drugs that block dimer formation, in essence to identify allosteric inhibitors of the receptor complex formation. However, Milligan and associates [121-122] developed an assay to identify compounds that promote dimer formation and that can detect agonists at the dimer. For their approach, they developed GPCR-G protein fusion proteins to set up a defined receptor-G protein stoichometry. They introduced mutations in one monomeric GPCR-G protein that maintained ligand binding properties but not G protein activation. In the other GPCR-G protein pair, they introduced mutations that prevented GTP exchange following agonist activation. Each monomer alone was inactive. However, when the dimer formed, a fully functional receptor complex was formed.

This assay provides an approach to identify compounds selective for activating heterodimers. Compounds effective on the dimer but ineffective on the monomers might be considered dimer selective. Of course, the next problem would be to determine whether such compounds are effective on the native receptors expressed in native tissue.

In fact, while GPCR oligomerization has drawn much interest, a major question remains as to whether GPCR dimers form in native tissue. Studying oligomerization in native tissues has been difficult because of the low expression levels of receptors and dimers. However, recent studies by Albizu *et al.* [31] have attempted to directly test whether GPCR oligomers actually exist in real life (native tissue).

For their studies, they detected dimers using a FRET assay. The assay was dependent on the use of fluorescent ligands binding to the receptors. Specifically, agonists and antagonists were used with tags that contained either FRET donor or acceptors. The tags did not affect ligand binding appreciably. The binding of the ligands to monomeric receptors produced no FRET response. However, if dimers were present and the ligands with acceptor and donors were in close proximity a FRET response could be detected.

The assay system was developed and optimized using tumor cell lines expressing the cloned GPCRs. The authors conducted studies on the cloned vasopressin, oxytocin and dopamine D2 receptors. Interestingly, antagonists could produce strong fluorescent responses while agonists produced much weaker FRET responses. The diminished FRET responses were not due to the agonist causing dimer uncoupling. There may be a number of reasons or interpretations for the weak FRET response to agonist binding. However, the authors suggested that the weak FRET responses following agonist binding were due to negative cooperativity and that essentially one agonist molecule binds to a dimer at a time. In contrast, the strong FRET response found with antagonists is likely to reflect two antagonist molecules binding to a single dimer at a time.

Ultimately, the purpose of these studies was to test whether the FRET based assay could be employed to detect dimers in native tissue. For this, the investigators studied oxytocin receptors in mammary glands from lactating rats. They employed the same ligands as used for the studies on the cloned receptors and showed that antagonist binding yielded FRET responses and in contrast agonist binding yielded much weaker responses. These studies were interpreted as supporting the notion that oxytocin receptor dimers can be detected in native tissues.

However, it should be noted that the studies used tissues that express extremely high levels of oxytocin receptor (1–3 pmol/mg protein), similar to the levels that may be normally expressed in tumor cell lines transfected with recombinant receptors. The investigators could not detect the dimers in tissues with lower receptor expression. In fact, using the same approach, they were not able to detect oxytocin or vasopressin receptor dimers in brain, a tissue with relatively robust receptor expression although at much lower levels than in mammary gland of lactating animals. Thus, the question remains whether dimers are naturally found in tissues in which receptor expression is not abnormally high. This issue is critical in determining the value of dimer selective drugs discovered in immortalized cell lines in which receptor expression is unnaturally high.

## 5. Novel Assays for GPCR Drug Discovery

Classical approaches to discover drugs targeting GPCRs have either employed radioligand binding assays, assays to measure GTPase activity or second messenger responses including changes in cAMP and Ca^++^ signaling [1,2]. Within the last decade, newer technologies have been developed and adapted for HTS that can allow for identification of novel types of GPCR drugs. These technologies include luminescent assays as well as label free technologies, some of which can now be employed to study GPCRs in native tissues.

### 5.1. Luminescent Assays for GPCR Drug Discovery

As described above, TR-FRET assays using ligands as acceptors and donors, and GPCR luminescent complementation assays and fluorescent receptor mutants can be employed to study dimer formation and pharmacology [31,113-115,120-122] and in a HTS format. Furthermore, for studies on the purified β-adrenoceptor, Kobilka and associates [3,4,6] were able to incorporate fluorescent probes into target sites within monomeric receptors to study conformational changes in the receptor in response to ligand binding.

This approach has been adapted to measure conformational changes in GPCRs in intact cells in a HTS format. This was done by developing FRET based assays in which donor and acceptor moieties were incorporated into the third intracellular loop and the C-terminus of receptors [123]. Under basal conditions in which the receptor is ligand free, energy exchange occurs between donor and acceptors in the different regions of the receptor. Agonist binding can promote a conformational change moving the donor and acceptor away for each other to reduce the FRET response. This may in fact be due to association of the receptor with G protein which would be expected to block acceptor-donor association.

This assay was validated in studies with the alpha_2_-adrenoceptor and muscarinic receptors [123,124]. Using these technologies, full and partial agonists can be clearly distinguished from antagonists. In fact, by generating profiles of ligand binding, one can use the technologies to sort out novel forms of ligand binding (ligands that induce conformational changes different than the known agonists or antagonists) providing a means to identify allosteric modulators. Importantly, this cell based assay is independent of signaling pathway, second messenger and G protein coupling to the GPCR and simply measures effects of ligand binding on receptor conformation.

### 5.2. Label Free Assays for GPCR Drug Discovery

Some of the label free technologies for GPCR drug screening provide complex phenotypic readouts and include DMR, described above, which uses resonant waveguide grating optical biosensors [87-89], impedance spectroscopy [125-127], and surface plasmon resonance [128,129]. These label free technologies use biophysical readouts to measure alterations in cell function. Their advantages over other optical technologies are that they do not require addition of dyes that can cause photobleaching or cellular transfection of recombinant proteins which by themselves can affect cell activity or other reagents. They also do not suffer from compound interference of the readout, as occurs with some calcium fluorescent assays and in some cases can be employed to study GPCRs in native tissues and under normal expression levels. This is particularly relevant as the pharmaceutical industry focuses more and more in using native tissues and endogenous receptors to discover drugs [130]. Generally these approaches are highly sensitive, require relatively few cells and can be employed in a HTS formats of 384 wells per plate or higher.

These technologies can distinguish different G protein signaling pathways for a specific GPCR, as shown in studies by Schroder *et al.* [89]. The readouts can be highly sensitive and provide measured responses in real time. Importantly for drug discovery and development, these technologies are amenable for structure activity relationship (SAR) analysis providing a means for standard drug development.

#### 5.2.1. Cellular impedance spectroscopy

Impedence spectroscopy measures changes in current flow through cells as a phenotypic response to drug action. The idea of this technology for use in cell biology is that when a drug, such as a GPCR agonist stimulates a cell in a selective manner, the resultant signal transduction can induce changes in cell morphology, volume, adherence and interaction with other cells. These alterations in the cell's biology affect current flow through the cell. Therefore, a change in current flow resulting from drug action will induce an alteration in impedance which is detected in the system.

To detect impedance changes, cells can be cultured either onto multielectrode arrays (MEAs) or they can be used in MDS Sciex (now Danaher) CellKey technology which consists of 96 well plates with each well containing gold electrodes at the bottom and fluidics and environmental control systems compatible with automated robotics systems. In these systems, alternating voltages are applied to the cells and the current measured. Calculation of the ratio of the voltage to current reveals the resultant impedance. The response of the cells to drug is detected as changes in the magnitude of the impedance.

The Cellkey technology has been employed to measure impedance responses of GPCRs, both endogenous and recombinant, to drug action. Verdonk *et al.* [125] employed the technology in panning studies using a variety of different immortalized cell lines. Panning studies involve screening a number of different GPCR ligands against the cells in an effort to identify endogenous receptors that are functionally active. The cellular dielectric spectroscopy response detected in these studies corresponded to what had already been known about the endogenous GPCRs found in these cells from other assays. Furthermore, the pharmacological profile of drugs tested against the receptors was similar to assays that measured intracellular signaling molecules such as cAMP or Ca^++^. The technology was sensitive enough to detect partial agonists and could be employed to determine antagonist affinities using classical Schild analysis. This is of importance since the endogenous receptors detected are generally expressed at low levels, consistent with might be found in primary cells. This finding suggested that the technology is adaptable to drug screening against primary cells and likely differentiated cells derived from embryonic stem cells. Interestingly, like the studies using DMR, the impedance assay could distinguish responses dependent on G protein coupling, that is activation of receptors coupled to different GPCRs gave different patterns of impedance responses.

The use of the CellKey technology for GPCR drug discovery was validated by a group at AstraZeneca [126]. They found the technology had high precision, reproducibility, and throughput needed for standard use in drug discovery with a large signal window and dynamic range allowing for structure-activity relationship studies. Furthermore, results where compatible with similar studies using either [^35^S] GTPgammaS binding or cAMP accumulation. The technology was also amenable for detection of positive allosteric modulators, which generally are difficult to detect with most standard GPCR assays, especially for endogenous GPCRs.

Importantly, in follow up studies, Peters *et al.* [127] compared the CellKey impedance assay with the DMR technology for detecting GPCR activation. They found the technologies were generally similar in their ability to detect receptor activation and pharmacology and indicated they were both able to detect inverse agonism. They concluded that both technologies were useful for drug discovery with many advantages over the more classical approaches of measuring receptor pharmacology.

#### 5.2.2. Surface plasmon resonance

Like DMR and impedance spectroscopy, surface plasmon resonance (SPR) is a label free, non-destructive optical technology that has been primarily used to measure binding kinetics of different biomolecules, in particular DNA-DNA, protein-protein and DNA-protein binding but is also beginning to find applications in measuring functions of intact cells and has been employed to study GPCRs [128,129]. The technology basically detects changes in the refractive index at the surface of an SPR biosensor as a measure of binding of biomolecules to that surface. Thus, when light is reflected from a biosensor at an angle greater than the critical angle, photons from the light interact with surface plasmons to cause a reduction in the intensity of the reflected light. The amount of photons absorbed by the bound surface molecule is directly related to the change in reflected light intensity providing a way to quantify the mass of the molecule bound to the biosensor surface. For example, if a cell expressing a specific antigen binds to an antibody on the surface of a SPR sensor, the mass change of the cell attached to the surface is detected as a change in the thickness of the adsorbed layer which is proportional to the reduction in reflected light intensity.

SPR has a number of advantages for use in studying GPCRs. First, the technology is extremely sensitive and is able to study femtomolar levels of receptor in lipid bilayers [128]. As a consequence, the assay can detect receptors in native tissues as a well as recombinant receptors. Furthermore, the technology can distinguish between mass and conformational changes in the receptor. Thus, it can establish whether ligand binding may recruit other molecules to receptors or change the conformation to affect G protein activation. In this respect, SPR does have a disadvantage in that it is primarily employed for study of GPCRs that bind endogenous peptides or proteins, because the mass shift of small molecule binding is relatively small and consequently the sensitivity of the technology at times may not be great enough to detect such small changes.

Studies by Alves *et al.* [129] focused on the peptide delta opioid receptor and used SPR as a response to ligand binding. Importantly, the spectral shifts induced by peptide and non-peptide agonists clearly differed as did antagonist and inverse agonist binding. Thus, by profiling different ligands, one can employ SPR to identify novel ligand binding, such as might be expected to occur with allosteric modulators. SPR has also been employed to measure conformational changes in opiate, beta-adrenergic and cannabinoid receptors in membrane preparations.

More recently SPR has been employed to study cells. One of the first examples of its use to study cells was described by Peterson *et al.* [131] who used SPR to study the release of fibronectin from vascular smooth muscle cells as the cells developed an extracellular matrix. The approach was highly sensitive, being able to detect as little as 20 ng of protein release in a 20 cm^2^ area.

In addition to SPR, use of nanoparticles to measure plasmon resonance with optical techniques has been recently employed to detect molecular events in single cells. Thus, Jun *et al.* [132] have used 40 nm gold nanoparticles, which they refer to as plasmon rulers, to measure biomolecular interactions using a similar concept as SPR. The nanoparticles can scatter light and can act as plasmons. The wavelength of the reflected light can change as a function of the distance of the particle to other gold particles and therefore can be used to measure biomolecular interactions. Consequently, these studies suggest the SPR might be employed to study GPCRs in intact cells as a method to measure novel ligand binding.

## 6. Conclusions

Basic research is driving the development of entirely new fields of GPCR drug discovery and development. It is providing the basis for discovery of compounds with new mechanisms of action, greater GPCR selectivity and unique biological actions. The hope is that these new generations of compounds can be translated into drugs with distinct and desirable therapeutic properties.

First and foremost is the development of allosteric ligands as designer drugs. Physical analysis of GPCRs by crystallography and NMR not only provides insight into the mechanics of drug-receptor interaction but yields critical information on how to rationally design allosteric modulators at specific receptors. We can begin to pick out specific sites on a receptor to affect receptor activation to produce designed responses, such as affecting one signaling pathway over another or to produce activation without desensitization or to facilitate activation only when the endogenous transmitter is present but not when it is absence to produce discrete signaling through the GPCR. The physical and computational analysis allows one to select compounds with particular chemistries to interact with selected sites on the receptor. The new screening technologies provide approaches to select for allosteric modulators using pharmaceutically user friendly HTS assays. Some of these technologies allow for phenotypic responses in native tissues to identify compounds more likely to produce desired therapeutic effects than assay systems dependent on immortalized cell lines over-expressing the receptors. Thus, one would predict a shift in direction of GPCR drug discovery to identification of new allosteric modulators discovered against endogenous GPCRs which may provide more interesting and potentially useful drugs than found with more classical orthosteric ligands.

Drug discovery against dimers also holds promise for the development of unique pharmacological agents and drugs. This may be particularly the case if the dimers have ligand binding properties distinct from the monomers. In this regard, heterodimer drug discovery may provide an approach to develop drugs that are region or tissue selective, which is presently not easily accomplished with classical drug discovery approaches. Furthermore, because dimers are predicted to be in equilibrium with monomers, drugs targeting dimers may shift the equilibrium thereby increasing the biological impact of dimer selective drugs. However, before the full potential of dimer pharmaceutics is realized, much more basic research will be necessary to establish the physiological role of these complexes *in vivo*. A major hurdle in this effort has been the problem of sensitivity, that is, the assays to detect dimers in physiological systems is still not adequate. This is due to the generally low level of endogenous expression of dimers in non-artificial systems. As the technologies improve, it is likely the evidence for unique dimers in brain and other organs will increase as will the interest and necessity to focus on these proteins for drug discovery.
